# DC current–voltage and impedance spectroscopy characterization of *n*CdS/*p*ZnTe HJ

**DOI:** 10.1038/s41598-024-63615-6

**Published:** 2024-06-05

**Authors:** I. Lungu, R. E. Patru, A. C. Galca, L. Pintilie, T. Potlog

**Affiliations:** 1 Laboratory of Organic/Inorganic Materials for Optoelectronics, Moldova State University, 2009 Chisinau, Republic of Moldova; 2 Complex Heterostructures and Multifunctional Materials Laboratory, National Institute of Materials Physics, 077125 Magurele, Ilfov Romania

**Keywords:** CdS/ZnTe heterojunction structures, Current–capacitance–voltage characteristics, Impedance–temperature–frequency characteristics, Electrical and dielectric properties, Applied physics, Energy science and technology, Materials science

## Abstract

This paper describes the electrical and dielectric behavior of the *n*CdS/*p*ZnTe HJ by current–voltage, capacitance–voltage characteristics, and impedance spectroscopy in a temperature interval 220–350 K. A microcrystalline *p-*ZnTe layer and *n*-CdS were grown on glass/ZnO substrate by closed space sublimation method. As frontal contact to CdS, the transparent ZnO and as a back contact to ZnTe, silver conductive paste (Ag) treated at 50 °C in vacuum were used. The current–voltage results of *n*CdS/*p*ZnTe HJ show a rectifying behavior. The junction ideality factor, barrier height, and series resistance values were extracted from the rectifying curves at different temperatures. The built-in voltage, carrier concentration and depletion width were obtained from the capacitance–voltage measurements. Analysis of the J–V–T and C–V–T characteristics shows that the thermionic emission and recombination current flow mechanisms dominate in the nCdS/pZnTe HJ. The dielectric study reveals that the experimental values of the AC conductivity, dielectric constant, dielectric loss, the imaginary part of the electric modulus are found to be very sensitive to frequency and temperature. The dielectric constant and dielectric loss are observed to be high at the low frequency region. The increase in the values of electric modulus with the frequency implies an increase in the interfacial polarization at the interface of *n*CdS/*p*ZnTe HJ. Jonscher’s universal power law shows that with increasing frequency, AC conductivity increased. The results conductivity show that the ionic conductivity and interfacial polarization are the main parameters affecting the dielectric properties of the device when the temperature changes.

## Introduction

The A_2_B_6_ semiconductor compounds possess excellent material properties, especially for photovoltaic (PV) applications. It is difficult today to imagine solid state devices without semiconductor heterostructures. Electronic devices, widely used in many areas of human activity, for example, in telecommunication systems, high-frequency devices, and satellite television systems, are difficult to imagine without the use of heterostructures. Scientific interest in A_2_B_6_ heterostructures are due, on the one hand, to their non-trivial physical properties associated with a complex ordered structure and interaction at the interface, and on the other hand, the prospect of their use in the formation of the effective photodevices. All semiconductors of A_2_B_6_ group, except for CdTe, have monopolar conductivity: ZnS, ZnSe, CdS, CdSe—*n-*type, ZnTe—*p*-type^1^. Recently, heterojunctions based on CdTe thin films are mainly used in photovoltaics. As CdTe PV cells reached commercialization, questions arose about potential cadmium emissions from CdTe PV modules. Some have criticized CdTe photovoltaic technology as inherently polluting and compared hypothetical emissions of cadmium from photovoltaic modules with emissions from coal-fired power plants. To overcome the challenges in the conventional CdTe-based solar cells, several researchers have reported ZnTe-based solar cells. ZnTe semiconductor has been of interest for solar cells owing to its wide direct bandgap of 2.24 eV at room temperature^1^, high electronic affinity of 3.73 eV^2^, high absorption coefficient of 10^5^ cm^−1^^3^ and high potential conversion efficiency with low-cost production and concern over environmental effects. ZnTe crystallizes in zinc-blend crystal structure and its conductivity is *p*-type doping due to native defect structure, such as zinc vacancy^4–6^ in contrast with other wide-bandgap semiconductors such as ZnO, ZnS materials typically* n*-type and difficult to convert to *p*-type conduction. In this paper, it is suggested to substitute widely used CdTe semiconductor absorber with *p-*ZnTe^6^ and investigate the electrical and photoelectrical properties of *n*CdS/*p*ZnTe heterojunctions.

## Experimental

**Figure 1:**
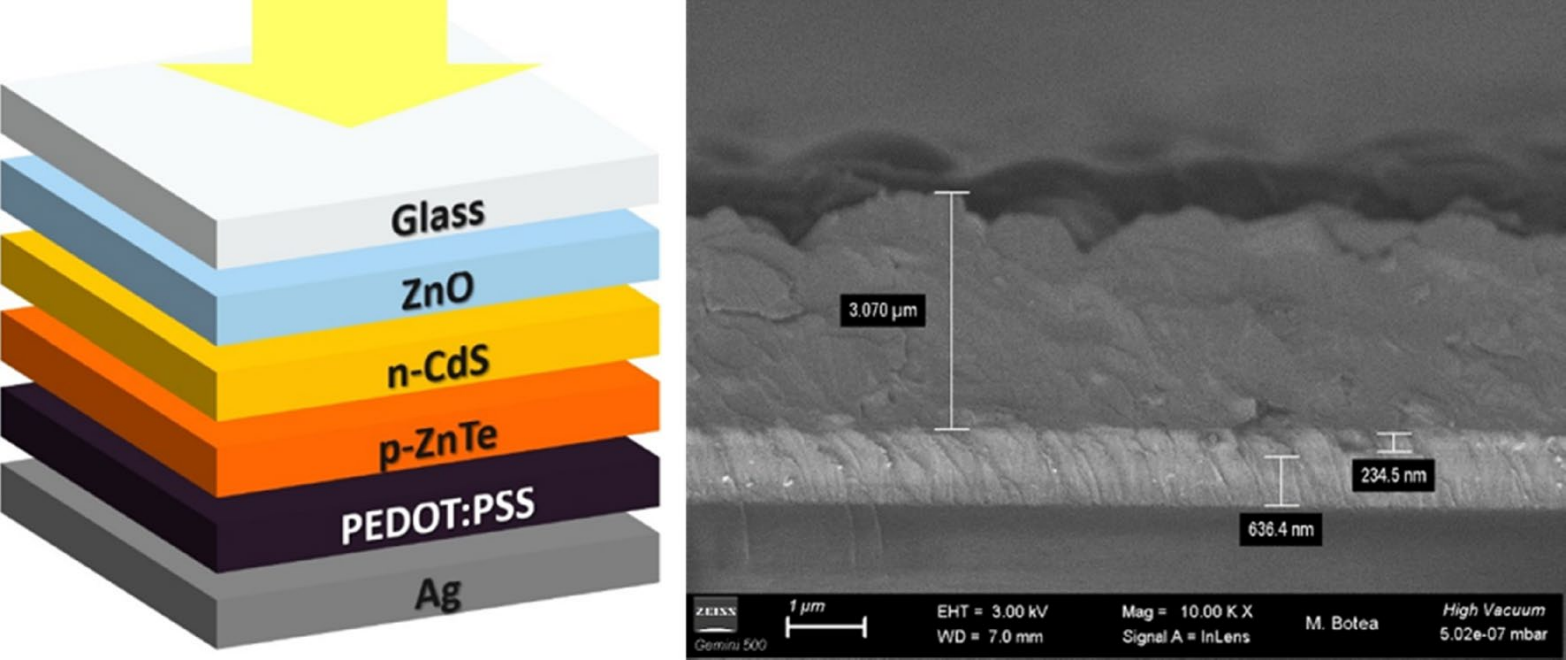
Schematic image of the fabricated (left) and cross-section (right) image of an *n*CdS/*p*ZnTe heterojunction.

The fabrication of *n*CdS/*p*ZnTe HJ involved several steps. The transparent electrode ZnO:Al:Cl thin films with a resistivity of 7 × 10^–4^ Ω cm, transparency of 85%, conductivity of approximately 1316 (Ω cm)^−1^, and a concentration of 1.25 × 10^21^ cm^−3^ were deposited on glass substrates using DC magnetron sputtering^7^. A CdS window layer with a thickness of ~ 240 nm and a ZnTe absorber film of varying thicknesses were grown by closed space sublimation (CSS) method using high purity (99.999%) CdS and ZnTe powders purchased from the German Company Alfa Aesar^6^. The deposition of the ZnTe thin film was carried out at T_sub_ = 340 °C and T_S_ = 600 °C. After ZnTe absorbent layer deposition, a buffer layer of PEDOT: PSS by drop casting, on the surface was synthetized. This strategic addition of PEDOT:PSS layer played a crucial role in optimizing the value of the open circuit voltage. As a back contact for optimized structures, silver conductive paste (Ag) treated at 50 °C in vacuum for 30 min was used. The schematic structure is shown in Fig. 1.

DC current–voltage characteristics were performed using a Keithley 2400 source meter. AC impedance measurements were carried out in vacuum, using an HIOKI 3532-50 LCR impedance analyzer at frequencies ranging from 10^2^ to 10^6^ Hz, temperature interval from 220 to 350 K, by applying a sinusoidal voltage having 0.2 V.

## Results and discussions

### The current–voltage and capacitance–voltage characteristics

**Figure 2:**
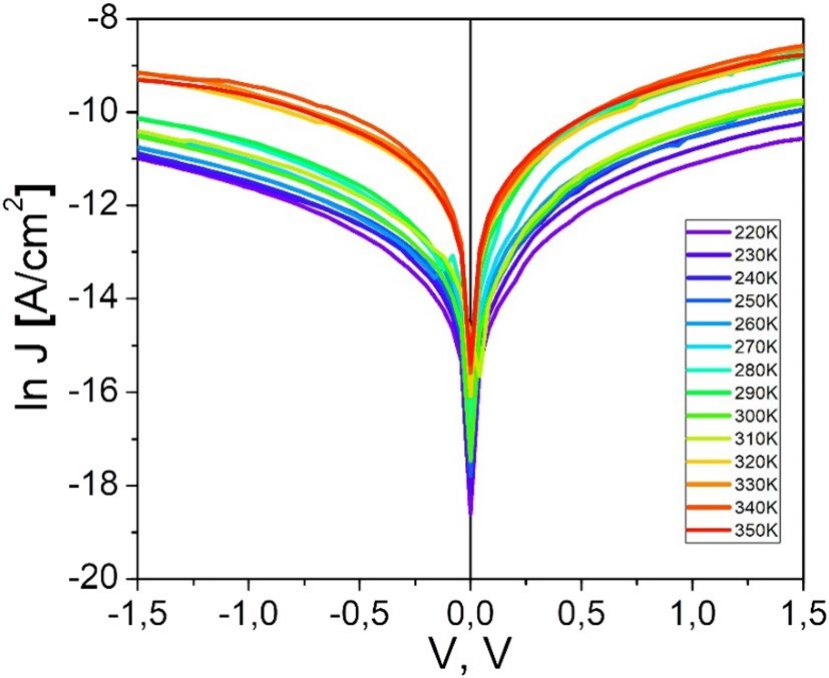
Dependence ln *J* = f(*V*) of the *n*CdS/*p*ZnTe HJ at different temperatures.

The most important electrical parameters of *n*CdS/*p*ZnTe HJ were determined by the current–voltage and capacitance–voltage (C–V) characteristics at different temperatures. From the current–voltage (J–V) curves when measured in the dark, the important parameters are the saturation current density (*J*_0_), ideality factor (*n*), series resistance (R_S_) and shunt resistance (*R*_*sh*_). Figure 2 shows the measured dark J–V–T characteristics symbols of the *n*CdS/*p*ZnTe HJ in a semi-logarithmic scale within the temperature interval of (220–350) K. The J–V characteristics can be described as follows:

1 $$J={J}_{0}exp\left(\frac{qV}{nkT}\right)\left[1-exp\left(\frac{-qV}{kT}\right)\right],$$

where *J*_*0*_ is the saturation current, *q* is the electron charge, *V* is the applied voltage across the junction,* n* is the ideality factor, k is Boltzmann’s constant, T is the absolute temperature in Kelvin. The saturation current density in a Schottky diode with a relatively low doped semiconductor (ZnTe with N_A_ ~ 10^15^ cm^−3^) is given by:

2 $${J}_{0}=A{A}^{**}{T}^{2}exp\left(-\frac{q{\Phi }_{b0}}{kT}\right),$$

where A** = 4πqm*k^2^/*h*^3^ is the effective Richardson constant, for ZnTe m* = 0.6 m_0_^8^. *A* is the diode area of the contact, *qΦ*_*b0*_ is the Schottky barrier height at zero voltage. The barrier height *qΦ*_*b0*_ are calculated from the equation:

3 $${\Phi }_{0}=\frac{kT}{q}ln\left(\frac{A{A}^{**}{T}^{2}}{{J}_{0}}\right).$$

The rectification coefficient of *n*CdS/*p*ZnTe HJ, at ± 1 V, changes in the temperature interval (300–350) K from ~ 5 to ~ 10. The extrapolated current from the forward ln J = f(V) region to V = 0 gives the value J_0_ presented in Table 1. The ideality factor was estimated from the slopes of the linear regions of the plot of natural log of forward current versus forward voltage using equation:

**Table 1:** The saturation current density (*J*_0_), ideality factors (*n*_1_, *n*_2_), rectification coefficient (*K*), barrier height (*qΦ*_*b*_) and built-in voltage (*V*_*bi*_) of *n*CdS/*p*ZnTe HJ.

T	ln J_0_	J_0_, 10^–5^ A/cm^2^	n_1_ V < 0.2 V	n_2_ V > 0.2 V	K	*qΦ*_*b,*_ eV	V_bi_, V
220	− 12.0	0.62	1.76	20.78	1.0	0.69	0.43
230	− 11.8	0.75	1.34	15.63	1.6	1.1	0.69
240	− 11.6	0.91	0.83	13.33	1.7	1.26	0.79
250	− 11.4	1.12	1.71	13.82	1.5	1.34	0.84
260	− 11.2	1.37	2.41	13.18	1.8	1.17	0.73
270	− 11.0	1.67	2.56	11.98	2.7	1.31	0.82
280	− 10.8	2.04	2.08	11.88	3..9	1.39	0.68
290	− 10.6	2.49	1.96	10.95	4.2	1.01	0.63
300	− 10.4	3.05	2.18	10.67	4.8	1.39	0.87
310	− 11.2	1.37	1.97	12.05	5.4	1.61	1.01
320	− 9.8	5.56	1.96	11.09	6.3	1.15	0.72
330	− 9.4	8.30	1.81	12.49	7.6	1.15	0.72
340	− 9.6	6.79	1.60	11.33	9.4	1.33	0.83
350	− 9.5	7.50	2.01	12.10	10.1	1.61	1.01

4 $$n=\frac{q}{kT}\frac{dV}{d\left(lnJ\right)}.$$

It is observed that the ideality factor decreased from 20.78 (220 K) to 11.88 (280 K), while the build in voltage and the saturation current density increases with increasing temperature.

The electrical parameters estimated from the J–V dependencies at different temperatures are presented in Table 1.

The effective Richardson constant A** can be estimated from the relation:

5 $$ln\left(\frac{{J}_{0}}{{T}^{2}}\right)=ln\left(A{A}^{**}\right)-\frac{q{\Phi }_{b0}}{kT}$$

**Figure 3:**
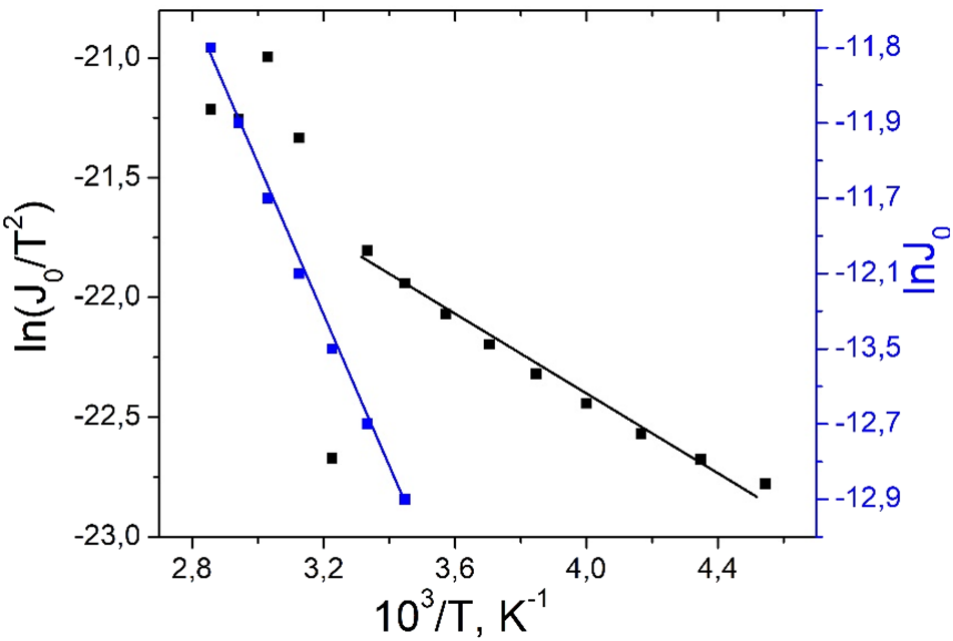
The ln(J_0_/T^2^) vs 1000/T plot for *n*CdS/*p*ZnTe HJ.

When ln (J_0_/T^2^) = f(1/T) is plotted for the (280–220) K temperature interval (Fig. 3), a straight line is observed. From the slope ln(J_0_/T^2^) versus 1/T dependency and from the intercept at an ordinate of the respective linear slope, the activation energy (*E*_*a*_) and the effective Richardson constant (A**) values were obtained as 0.98 eV and 69 A/cm^2^K^2^, respectively. The estimated effective Richardson constant slightly deviates from the theoretical value presented in the publication^9^. The barrier height in the temperature region (220–280) K was changed from 0.69 eV (220 K) to 1.01 eV (290 K), while the ideality factor varied from 20.78 to 10.95, respectively. For an ideal diode structure, the barrier height should increase as temperature is decreased, in accordance with the band gap variation with temperature^10^. According to the Table 1, the barrier height shows an inverse behavior to the ideality factor variation. The ideality factor increases, while the barrier height decreases with decreasing temperature. The increase in the value of the barrier height at high temperatures can be correlated with the modification of the concentration of free carriers at the Ag/*p*ZnTe interface induced by the temperature. In addition, the increase in the value of barrier height with increasing temperature to 280 K can be explained by the reduction of the carrier concentration in the depletion region of *n*CdS/*p*ZnTe HJ through the appearance of traps and recombination centers associated with high temperatures. With the further increasing of the measurement temperature, the barrier height first decreases, then it started to increase–decrease.

The decrease-increase of the ideality factor with increasing temperature is due to the inhomogeneity of barrier height. Probably, the changes in the values of the ideality factor indicate that the different recombination current losses affect performance of the device, such as interfacial recombination, bulk recombination, etc. Similar trends have already been reported by other authors^11,12^ and have been explained by assuming inhomogeneities at the interface. In addition, the values of barrier height and ideality factor measured at the *n*CdS/*p*ZnTe HJ in the high-temperature region from 290 to 350 K exhibited a deviation from the thermionic emission model. This could be attributed to the non-uniformity of the interfacial charges or the dislocations ^13,14^. The ideality factor values in the (290–350) K region have changed from 1.96 (290 K) to 2.01 (350 K). These features allow us to suppose that, under forward bias, at higher temperatures, the current transport mechanism is dominated by recombination at the interface. Normally volume impurities, surface imperfections, dislocations are the sites of recombination. So, the predominant current flow in the high temperature region, according to Fig. 3 is the recombination of the generated electron at surface imperfections and dislocations. Since the lattice constants of CdS and ZnTe differ by about 10%, this leads to the formation of broken bonds (surface states). It is well known^15^, that the concentration of mismatch dislocation is usually estimated as N_ss_ ~ *x*^−2^ in the first approximation are found to be equal to N_ss_ ~ 1/*x*^2^ ~ 6.12∙10^13^ cm^−2^, where *x-*the distance between the dislocations calculated as the difference of the two lattice constants values of the components of the HJ, *a*_*CdS*_ = 4.130 Å and *a*_*ZnTe*_ = 6.110 Å, divide by the average value of these two lattice constants. The high concentration of interface defects plays a role in carriers trapping or recombination, affecting the electric current transport mechanism. As forward bias is applied and the temperature increases, an enhancement in electron concentration at the interface results. These generated electrons recombine with the holes from the interface before they can be collected in the external circuit, leading to the decrease of photocurrent. From Fig. 3, at higher temperature > 290 K, another type of recombination takes place through defect (volume impurities) or dopant, known as Shockley–Read–Hall recombination that occurs via a trap level situated in the band gap, with *E*_*A*_ = 0.98 eV.

**Figure 4:**
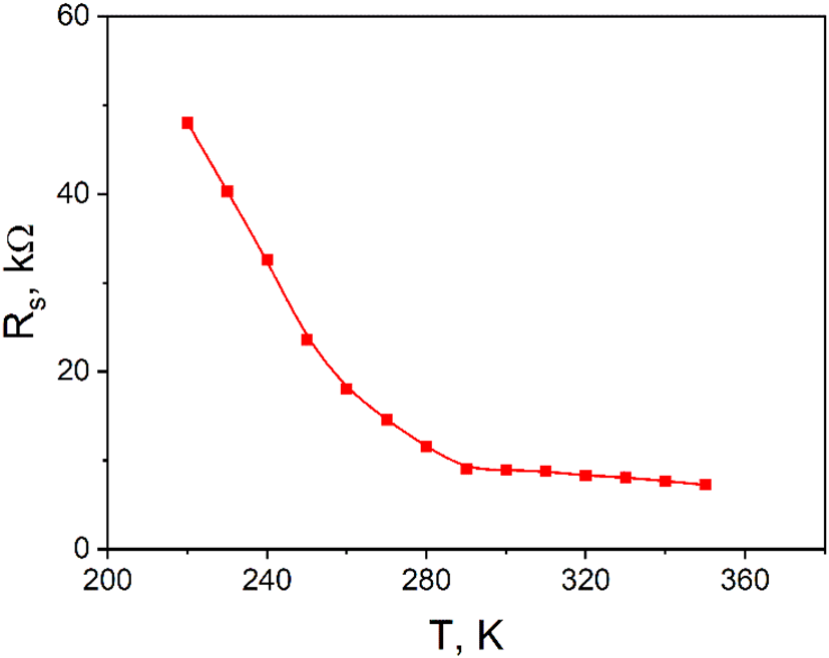
The temperature dependence of the *R*_*S*_ of the *n*CdS/*p*ZnTe HJ.

Furthermore, the high values of the ideality factor are attributed to the effects of the voltage drop across the interfacial layer and series resistance. We observed that an increasing voltage drop influences the J–V curves across the series resistance of the *n*CdS/*p*ZnTe HJ structure, thus dominating their behavior. In Fig. 4, the series resistance, estimated by extrapolating the dV/dJ characteristic to 1/J as 1/J → 0, is approximately 10 kΩ at room temperature and increases up to 60 kΩ at 220 K.

This effect is more pronounced at lower temperatures due to the increased resistivity of ZnTe with decreasing temperature. The decrease in series resistance is attributed to the increase in the concentration of charge carriers with increasing temperature^16^.

In the higher temperature region, the recombination component is more pronounced than thermionic emission. The component of the recombination at the interface is due to the lattice mismatch of CdS and ZnTe materials and recombination through energy state in the forbidden region unintentionally introduced into ZnTe during the manufacturing process is more evidenced that in the low temperature interval.

So, the forward current is dominated by thermionic emission at all temperatures and recombination via interface states and traps at high temperature prevails thermionic emission component.

**Figure 5:**
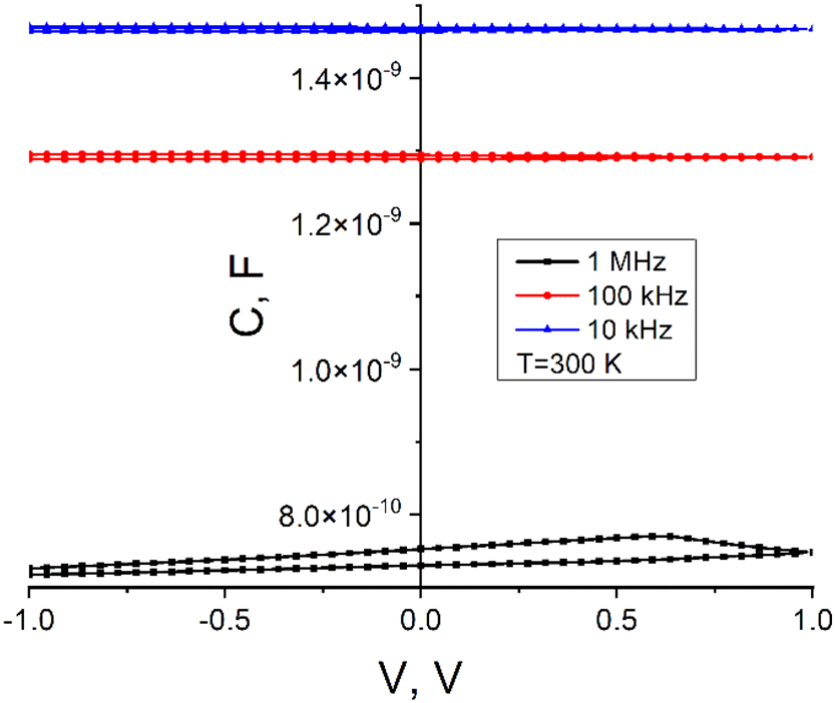
The C–V dependencies of the *n*CdS/*p*ZnTe HJ at different measurement frequencies.

More information about the built-in voltage, the impurity concentration in the constituent materials and the width of the space charge region is obtained from studying the capacitance–voltage (C–V) dependence. The measured C–V characteristics on the *n*CdS/*p*ZnTe HJ at different frequencies (10 kHz, 100 kHz, and 1 MHz) indicate hysteresis in the C–V curve. We choose a high frequency of 1 MHz to minimize the response of interface states. Under these conditions, the effect of interface states on the C–V response stretch out the C–V curve along the voltage axis. As the measurement frequency increases from 10 kHz to 1 MHz, the capacitance value of the *n*CdS/*p*ZnTe HJ decreases by almost an order of magnitude, suggesting the contribution of states at the heterostructure interface. Cyclical variation in the C–V characteristic occurs at all measurement frequencies (Fig. 5), and under all voltages, the junction capacitance decreases with increasing the value of frequency. The measurement temperature also minimally influences the cyclic variation of capacitance. At temperatures T > T_room_, the capacitance value increases, and the variation in capacitance with increasing temperature changes slightly. For example, it changes from ΔC = 0.004 F at T = 300 K to ΔC = 0.005 F at 350 K.

**Figure 6:**
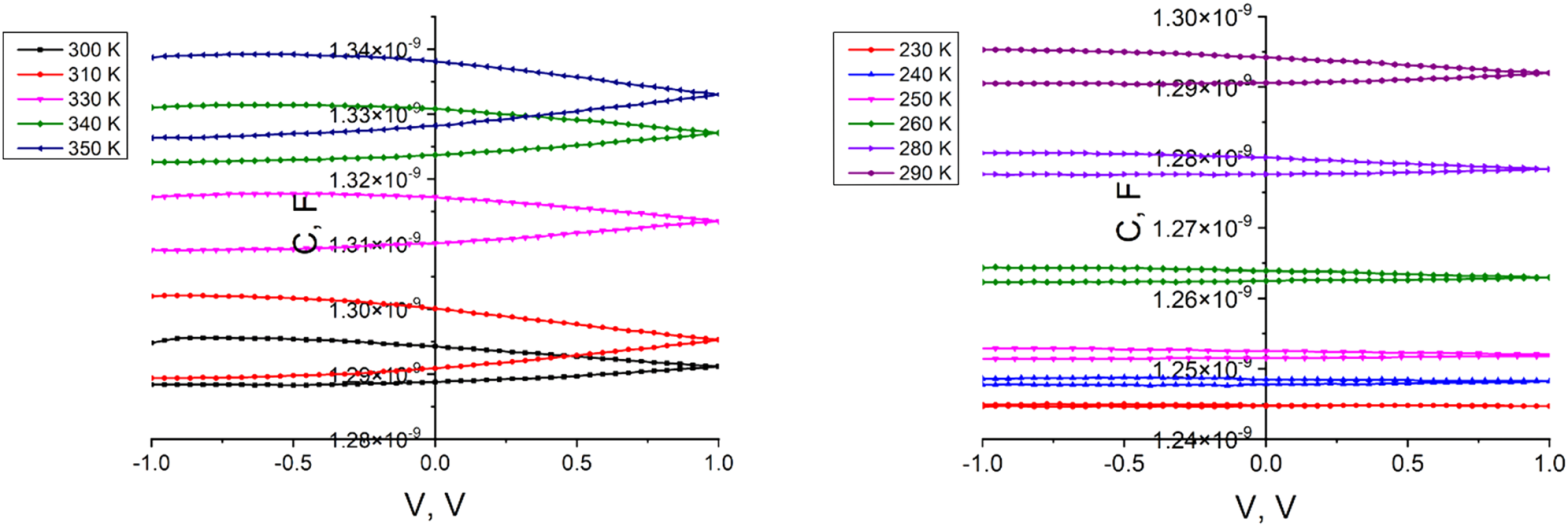
The C–V dependencies of the *n*CdS/*p*ZnTe HJ at different measurement temperatures (T > T_room_) (left) and (T < T_room_) (right).

At temperatures higher than room temperature, the C-V hysteresis appears much wider compared with that at room temperature and lower temperatures (Fig. 6). In Figs. 5 and 6, at + 1 V bias, the capacitance variation closes, while at negative biases of − 1 V, this feature is not observed. The observed hysteresis in the C–V curves suggests a high density of charge trapping centers.

**Figure 7:**
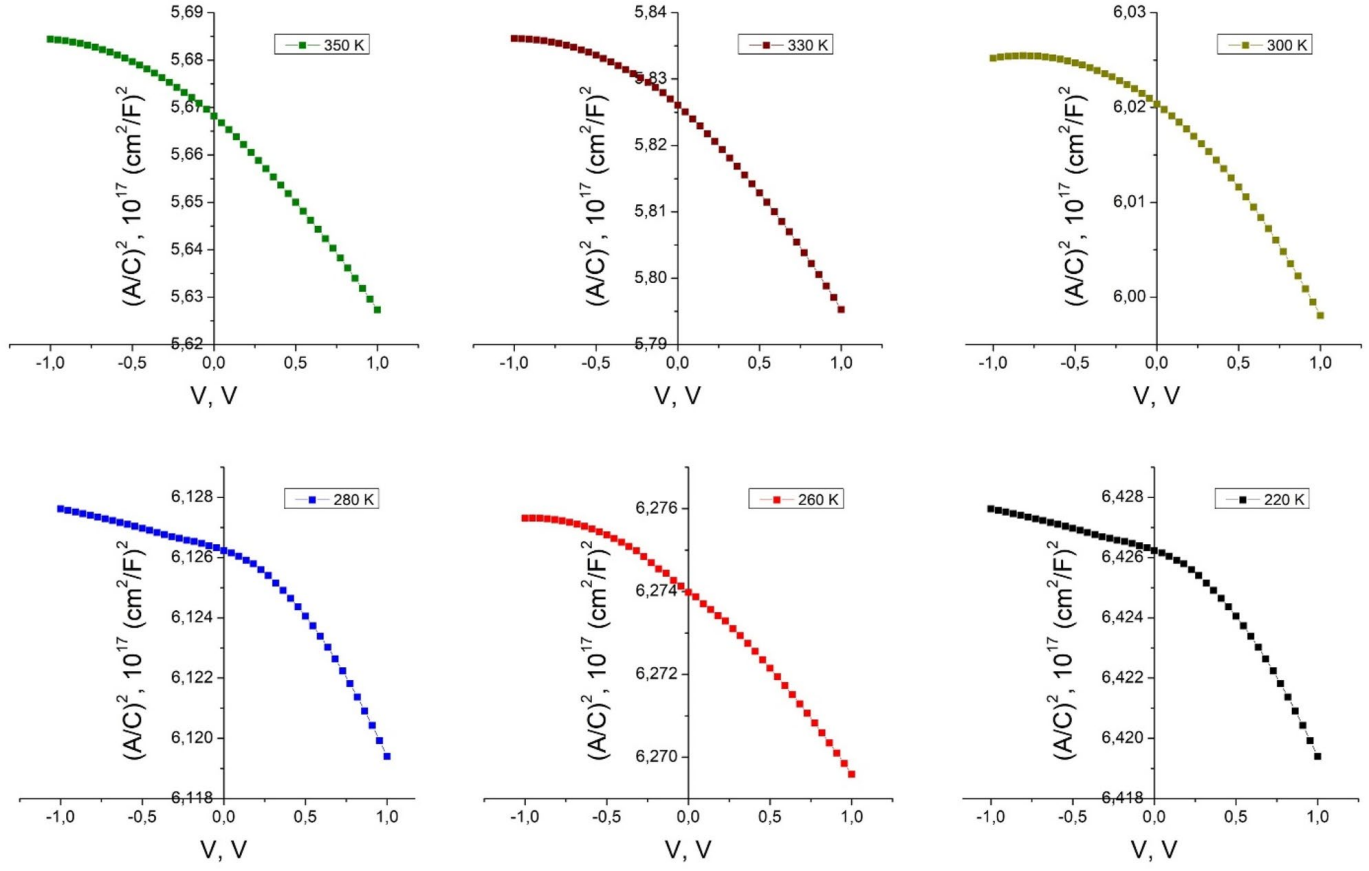
The C^−2^ = f(V) curves of *n*CdS/*p*ZnTe HJ for different temperatures.

The revealed C–V hysteresis could be attributed to the electron charging and discharging of the carriers by direct tunneling through the ultra-thin layer formed between the *n* and *p* films components of the heterojunction, which significantly affect the performance. The barrier height was determined from the 1/C^2^ = f(V) curves for different temperatures (Fig. 7). Mott–Schottky analysis allow to extract the doping density concentration and the built-in voltage using the relation:

6 $$\frac{1}{{{\text{C}}^{2} }} = \frac{2}{{{\text{A}}^{2} \varepsilon {\text{q}}}}\frac{1}{{{\text{N}}_{{\text{A}}} }}\left( {{\text{V}}_{{{\text{bi}}}} - {\text{V}}} \right),$$

where *ε* semiconductor permittivity, A is the device area, *q* is the unit charge, *N*_*A*_ is the doping density concentration in the bulk and *V*_*bi*_ is the built-in voltage. The capacitance dependencies from Fig. 7 show two slopes, one for negative voltages and another for positive voltages, suggesting that there are two different mechanisms contributing to the capacitance in these regions of voltages, that originates from different responses of trapped and mobile charges between the interface region and bulk of the semiconductor^17^. The shallow states respond to the high frequency voltages, while the deep and localized states do not respond to those voltages at high frequency^18,19^. Lower values of the capacitance at higher frequencies indicate that mobile charges do not respond at those high frequencies, behaving similar to fixed or trapped charges. Typically, the capacitance in the accumulation regime has shown a rapid decrease when the applied frequency starts to grow. This indicates that the capacitance of the structure to the signal above 100 kHz decreases rapidly. The capacitance value to the voltage signal is mainly constrained by large bulk resistance of ZnTe. In contrast, the depletion capacitance remains almost constant. Since holes are depleted in ZnTe, the whole device can be considered as a series of two capacitors, one from the insulator and the other from the depletion region, with no resistance which constrains the response of the charges along the capacitors^17^.

The high-low frequency capacitance method was used for the estimation of the interface state density (*N*_*ss*_). We selected a low frequency of 10 kHz and a high frequency of 1 MHz to obtain the value of the interface state density of the CdS/ZnTe HJ. The capacitance at a low frequency (*C*_*LF*_) can be determined by:

7 $${C}_{LF}={C}_{it}+{C}_{sc}.$$

Thus, the equivalent capacitance becomes the parallel connection of interface state capacitance *C*_*it*_ and space charge capacitance *C*_*SC*_:

8 $${\text{C}}_{\text{sc}}={\text{C}}_{\text{HF}}.$$

Substituting Eq. (8) into Eq. (7) results the interface state capacitance (*C*_*it*_) in terms of the measured low frequency and the higher frequency curves as:

9 $${\text{C}}_{\text{it}}={\text{C}}_{\text{LF}}-{\text{C}}_{\text{HF}}.$$

According to^20,21^, the variation of the interface state capacitance (*C*_*it*_) with frequency is described by the relation:

10 $${\text{C}}_{{{\text{it}}}} = \frac{{{\text{Aq}}N_{{{\text{ss}}}} }}{\tau }\frac{{{\text{arctan}}\left( {\omega \tau } \right)}}{\omega },$$

where *N*_*ss*_ is the density of interface states, *A* is the area, *ω* is the frequency and *τ* is the relaxation time of the interface state. The interface state density *N*_*ss*_ taken into consideration (7) can be written as:

11 $$N_{ss} = \frac{{C_{it} }}{qA} = \frac{{\left( {C_{LF} - C_{HF} } \right)}}{qA}.$$

The interface state density at the CdS/ZnTe interface estimated by (11) reaches the value of 4.9 × 10^10^ eV^−1^ cm^−2^. The C–V measurements allow us to also estimate the width of the space-charge region. The width of the space-charge region varies with the measurement frequency, ranging from 2.16 µm (10 kHz) to 4.33 µm (1 MHz). A value that changes slightly around the value of 1.2 V of the built-in voltage *V*_*b*i_ is observed in the whole interval of the temperatures. The density of acceptor states (which is just the doping density *N*_*A*_ acceptor doping concentration) estimated from the linear slope of the C^−2^ = f(V) dependencies (Fig. 7) reached the values of 0.81 × 10^17^ cm^−3^ (350 K), 1.22 × 10^17^ cm^−3^ (300 K) and 3.23 × 10^14^ cm^−3^ (260 K).

Thus, the C–V measurements exhibit a hysteresis loop due to the charging and discharging of traps at the *n*CdS/*p*ZnTe HJ interface. Following Fleetwood et al. publications^22,23^, it is suggested that 15 types of boundary traps located near the interface can interact with the substrate and could be responsible for the observed C–V hysteresis.

**Figure 8:**
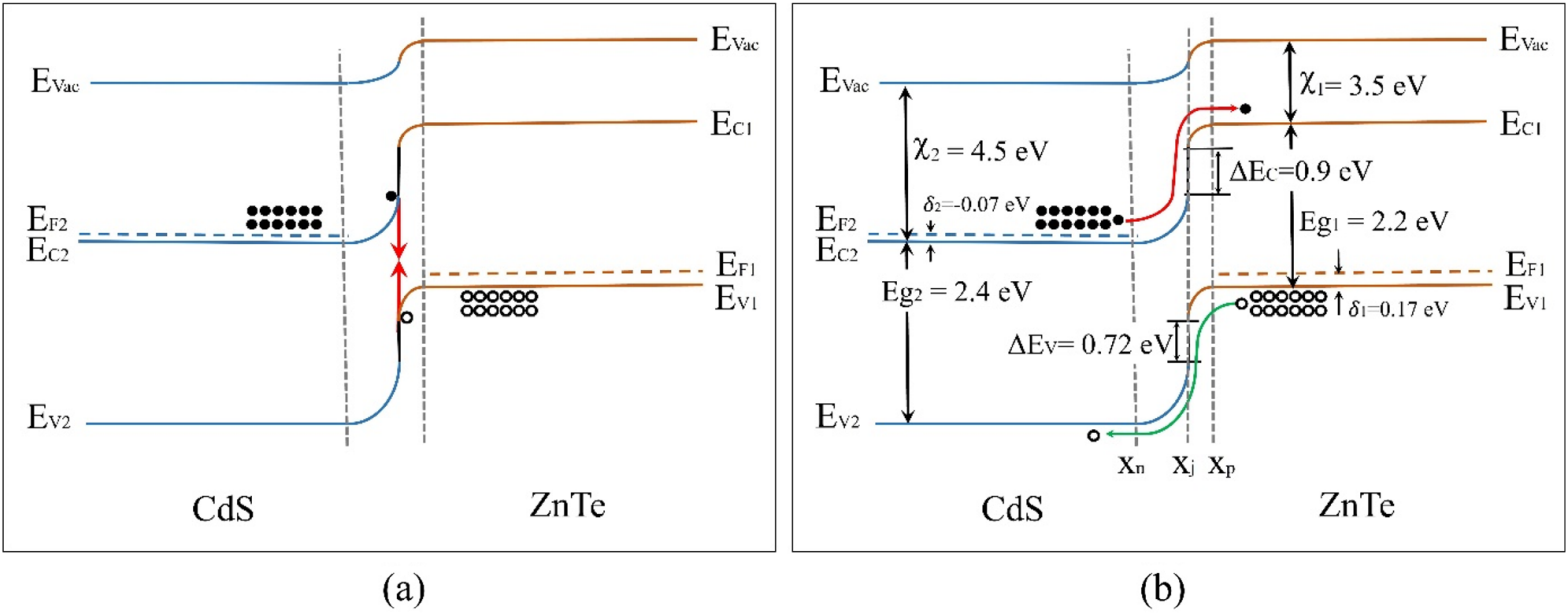
Energy band diagrams of the *n*CdS/*p*ZnTe HJ at a forward voltage below 0.2 V (**a**) and over 0.2 V (**b**).

Therefore, the good agreement between the predicted theory and experimental results from I–V and C–V characteristics confirms the proposed charge carrier transport model considered for describing the *n*CdS/*p*ZnTe HJ under forward bias (Fig. 8).

In addition, the formation and distribution of interface traps have been investigated through the study of complex dielectric permittivity and complex impedance.

### Dielectric properties

As the applications of electronic devices are significantly influenced by the dielectric properties of heterojunctions, in this study we will present the detailed analysis of the dielectric constant, dielectric loss, real and imaginary electric modulus, and AC electrical conductivity of the heterojunction by impedance spectroscopic technique. The ability of structures to accumulate the energy when interacting with an electric field of the form *E* = *E*_*0*_*e*^*(iωt)*^ (where *ω* represents the angular frequency, *E*_*0*_ is the field's amplitude, and* i* = (− 1)^1/2^, the dielectric permittivity can be represented by a complex function dependent on the frequency^24^:

12 $$\varepsilon = \varepsilon_{0} \varepsilon_{r} \left( \omega \right),$$

where *ε*_*r*_(*ω*) is the complex form of the relative dielectric permittivity, and *ε*_*0*_ represents the permittivity of vacuum. The relative dielectric permittivity is a complex quantity described by the following equation:

13 $$\varepsilon^{*} = \varepsilon^{\prime} - {\text{i}}\varepsilon^{\prime\prime},$$

where ε′ and ε″ are the real and imaginary parts of the complex dielectric permittivity, respectively.

It is well known that the total electric current density through a structure subjected to an electric field is determined by the formula:

14 $$\overrightarrow {{J_{T} }} = i\omega \varepsilon_{0} \left( {\varepsilon_{r} + \frac{\sigma }{{i\omega \varepsilon_{0} }}} \right)\vec{E},$$

where $\overrightarrow{{J}_{C}}$_=_*iω*ε_0_ε_r_ $\overrightarrow{E}$ represents the conduction current density, and—$\overrightarrow{{J}_{D}}={\varepsilon }_{r}{\varepsilon }_{0}\frac{\partial \overrightarrow{E}}{\partial t}$ represents the polarization current density.

Considering the total current density, the relative dielectric permittivity can be written in the form:

15 $$\varepsilon_{r} \left( \omega \right) = \varepsilon^{\prime}\left( \omega \right) - i\left( {\varepsilon^{\prime\prime}\left( \omega \right) + \frac{\sigma }{{\omega \varepsilon_{0} }}} \right).$$

The real component ε′ represents stored energy, while the imaginary component ε″ represents the dissipated energy. The imaginary part has two components as well: the first component, ε′(ω), is due to dielectric losses through the electrical conduction, and the second component, $\frac{\sigma }{\omega {\varepsilon }_{0}}$, is related to losses through polarization. Polarization processes are characterized by an exponential decay with a relaxation time τ and a critical frequency, *f*_C_ = 1/(2πτ), at which the highest energy loss, known as Debye relaxation, occurs.

The Debye equation for the complex dielectric permittivity is described by equation:

16 $$\varepsilon^{*} = \varepsilon^{\prime} - i\varepsilon^{^{\prime\prime}} = \varepsilon_{\infty } + \frac{{\varepsilon_{S} - \varepsilon_{\infty } }}{1 + i\omega \tau } - i\frac{\sigma }{{\omega \varepsilon_{0} }}.$$

From the Debye equation, the relations that describe the real and imaginary part of the complex dielectric permittivity components results:

17 $$\varepsilon^{\prime}\left( \omega \right) = \varepsilon_{\infty } + \frac{{\varepsilon_{S} - \varepsilon_{\infty } }}{{1 + \omega^{2} \tau^{2} }},$$

18 $$\varepsilon^{\prime\prime}\left( \omega \right) = \frac{\sigma }{{\omega \varepsilon_{0} }} + \left( {\varepsilon_{S} - \varepsilon_{\infty } } \right)\frac{\omega \tau }{{1 + \omega^{2} \tau^{2} }}.$$

**Figure 9:**
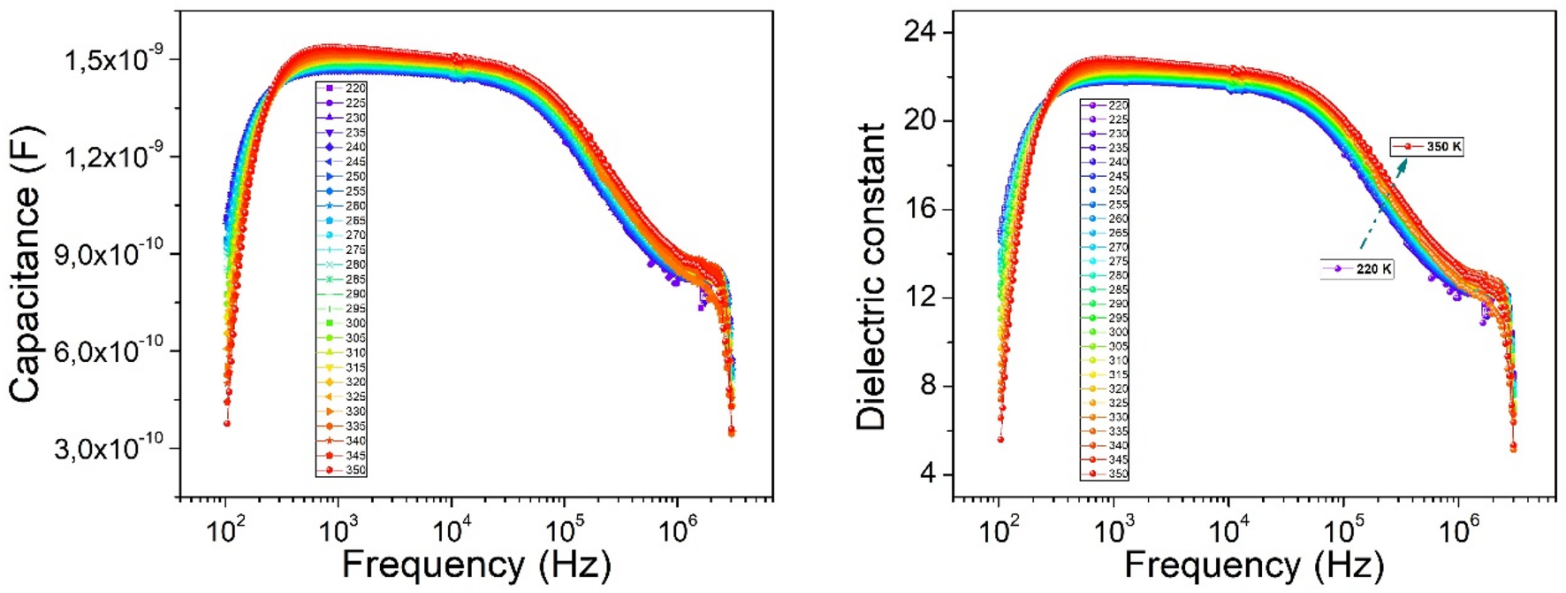
The C–F (left) and dielectric constant-frequency (right) dependencies of the *n*CdS/*p*ZnTe HJ at different temperatures.

ε_∞_ denoted the dielectric permittivity measured at the high frequency, and ε_s_ denoted the dielectric permittivity measured in the low frequency region. The ratio between the imaginary part of the complex dielectric permittivity and its real part is called the loss angle tangent or dissipation factor, tan*δ* = ε″/ε′. The loss angle tangent or dissipation factor (tan*δ*) was estimated directly from the measurement data. The measurements characteristics of capacitance as a function of frequency at different temperatures are presented in Fig. 9 (left). In this context, the capacitance is observed to remain relatively constant until (4–6)·10^5^ Hz, highlighting a plateau over this frequency region that signals dielectric relaxation. Afterwards, at frequencies higher than (4–6)·10^5^ Hz follows another type of dielectric relaxation. The notable importance is that the two periods of relaxation are the result of two distinct phenomena that have different origins. The dependence of the dielectric constant shown in Fig. 9 (right) determines the charge storage capacity in a dielectric material, which dictates the electric field distribution in the nCdS/pZnTe HJ, while the values of the dielectric constant indicate the possible dielectric losses in this one.

The dielectric constant decreases with the increasing of frequency due to the shrinking of space charge polarization effect and then, it is remained nearly constant with increasing temperature at the given frequency. An accumulation of charge at the grain boundary leads to higher values of the dielectric constant, while the presence of space charge polarization at the grain boundaries generates a potential barrier. The decrease of the real part of the dielectric constant at higher frequencies occurs because the structure of the dielectric is composed of grains with low resistivity separated by weakly conducting thin boundaries. When the electric field is applied, there is a localized accumulation of charges, which leads to interfacial polarization.

The capacitance and dielectric constant values of *n*CdS/*p*ZnTe HJ increase slightly with increasing temperature. The effect of temperature on the dielectric constant is like that of frequency. With increasing temperature, the mobility of polar molecules increases, which leads to an increase in the dielectric constant. For materials that possess permanent dipoles, a significant variation of the dielectric constant with temperature because of the orientation polarization is observed. First, the dielectric constant is strongly dependent on the structure of the dielectric. Secondly, the dielectric constant changes abruptly at the interface between the phases due to the fact that the structure changes at the phase change. So, depending on the phases involved, the dielectric constant increases or decrease at a certain phase change.

**Figure 10:**
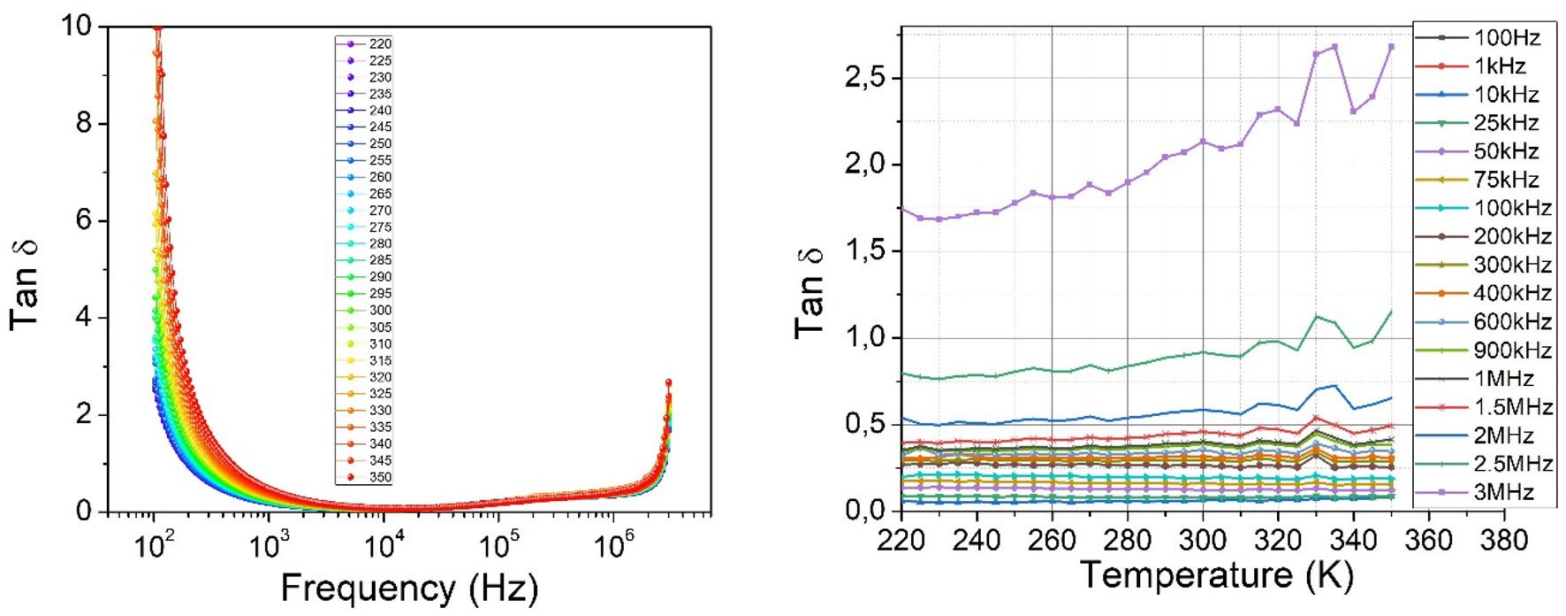
The frequency dependencies of dielectric loss of the *n*CdS/*p*ZnTe HJ for different measurement temperatures (left) and the temperature dielectric loss for different frequencies (right).

**Figure 11:**
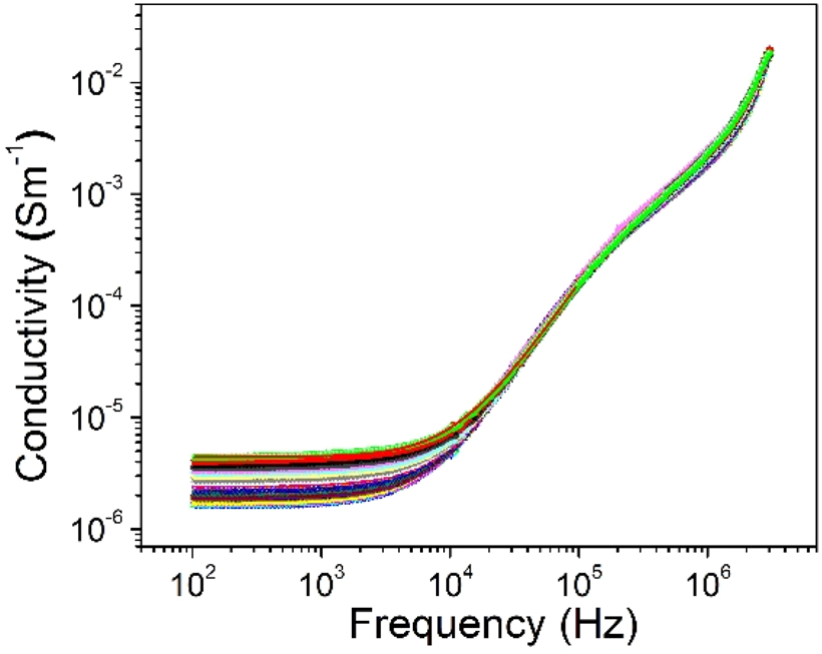
The total conductivity–frequency dependencies of the *n*CdS/*p*ZnTe HJ at different temperatures.

The dependence of the dielectric loss on the frequency of the electromagnetic field is complex. It can be seen from Fig. 10 (left), that with the increasing of the frequency, dielectric losses decrease, at studied temperatures. This phenomenon is attributed to the decrease in the degree of orientation of the electric dipole moment of the molecules. However, starting from a value of about 10^3^ kHz, dielectric losses remain constant. The increase in the dielectric loss is more pronounced at 2 MHz. It is important to note the fact that the presence of a different nature of dielectric relaxations in the *n*CdS*/p*ZnTe HJ is observed. As can be seen in Fig. 10 (right), the dielectric loss slightly increases with increasing temperature. The mobility of charge carriers increases with temperature due to increases the polarization and leads to high dielectric loss. Knowing the loss factor and dielectric constant we estimated the polarization conductivity of *n*CdS/*p*ZnTe HJ. In terms of total conductivity, Jonscher^25^ provided a detailed explanation of this phenomenon:

19 $$\sigma = \sigma_{{{\text{dc}}}} \left( {\text{T}} \right) + \sigma_{{{\text{ac}}}} \left( {\omega ,{\text{T}}} \right).$$

where σ_dc_(T) represents the electric conductivity, and σ_ac_(ω, T) represents the polarization conductivity. The variation of the total conductivity σ with frequency is illustrated in Fig. 11. It can be seen at low frequencies, a frequency-independent behavior is observed, corresponding to electric conductivity, while at high frequencies the polarization component of conductivity prevails. The temperature dependence of the total conductivity σ, shown in Fig. 11, suggests that σ_ac_ changes with temperature. The polarization conductivity is slightly influenced by temperature at higher frequencies.

20 $$\sigma_{{{\text{ac}}}} = {\text{A}}\omega^{{\text{s}}} ,$$

where: *A* represents a constant, *ω* is the frequency, and *s* is an exponent that generally has a value less than or equal to one.

**Figure 12:**
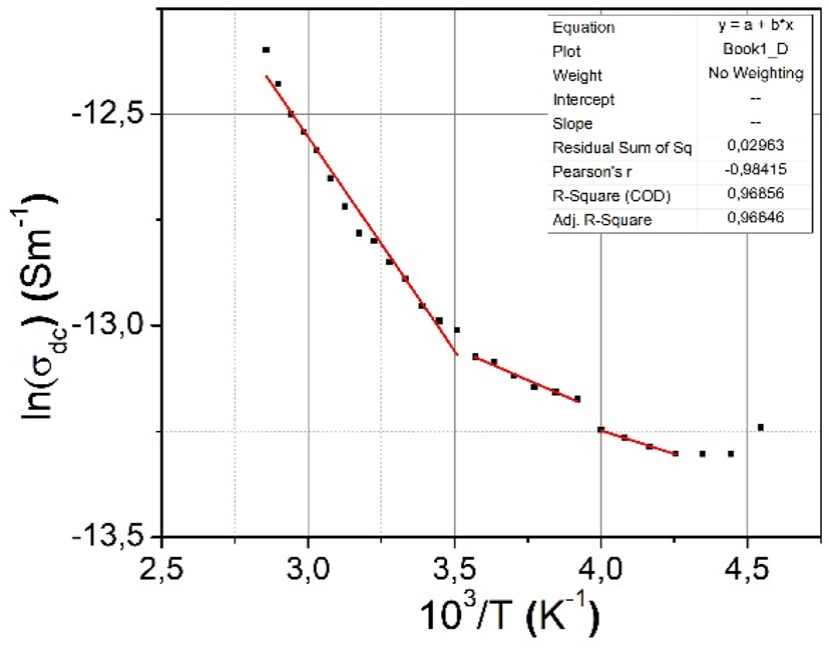
The conductivity-temperature dependence of the *n*CdS/*p*ZnTe HJ.

The value and behavior of the "*s*" exponent in the (18) determines the predominant conduction mechanism in the material. In dependence of the value and behavior of "*s*", several theoretical models have been developed to explain the conduction mechanism of materials (QMT, SPT, LPT and CBH). Within the quantum-tunneling model (QMT)^26^, it is suggested that "*s*" is influenced more by frequency but not by the temperature. In the case of the small polaron tunneling (SPT) model^27^, "*s*" is predicted to increase as the temperature increases. For large polaron tunneling (LPT)^28^, "*s*" depends on both temperature and frequency. In the CBH (correlated barrier hopping) model, where the conduction phenomenon derives from the synchronized hopping of electric charge carriers between nearest neighbor states^29^, "*s*" is assumed to depend on both, temperature and frequency, and "*s*" should decrease as the temperature increases. In the conductivity–frequency dependencies, two distinct regions can be observed near the frequency of 100 kHz, where a dielectric relaxation in the conductivity was noted. At high frequencies, the conductivity increases significantly as the frequency increases, suggesting that it tends to approach a dielectric conductivity around 100 kHz. At this point, dielectric relaxation takes place leading to a new phase of sharp decrease in conductivity, and it becomes constant at frequencies below 100 kHz. By analyzing these three regions shown in the measured dependence at different temperatures, according to Jonscher's law, we determined that the variation of conductivity as a function of frequency proceeds according to an "*s*" parameter of 1.63 at low frequencies and 2.7 at high frequencies. As can be seen in Fig. 12, the relationship between conductivity and temperature shows a linear variation in the (280–350) K and in (280–220) K temperature regions two other linear slopes that could not be explained by theory of the dc conductivity.

The behavior of the conductivity of the *n*CdS/*p*ZnTe HJ in the (280–350) K suggests that it is similar to a ferroelectric crystal ionic conductivity described by the equation:

21 $$\sigma_{{{\text{dc}}}} = \sigma_{0} \exp \left( { - \frac{{E_{{\text{a}}} }}{kT}} \right).$$

The activation energy *E*_*a*_ values in the (280–350) K are found as *E*_*a*_ = 0.86 eV.

### Electrical impedance analysis of the nCdS/pZnTe HJ

**Figure 13:**
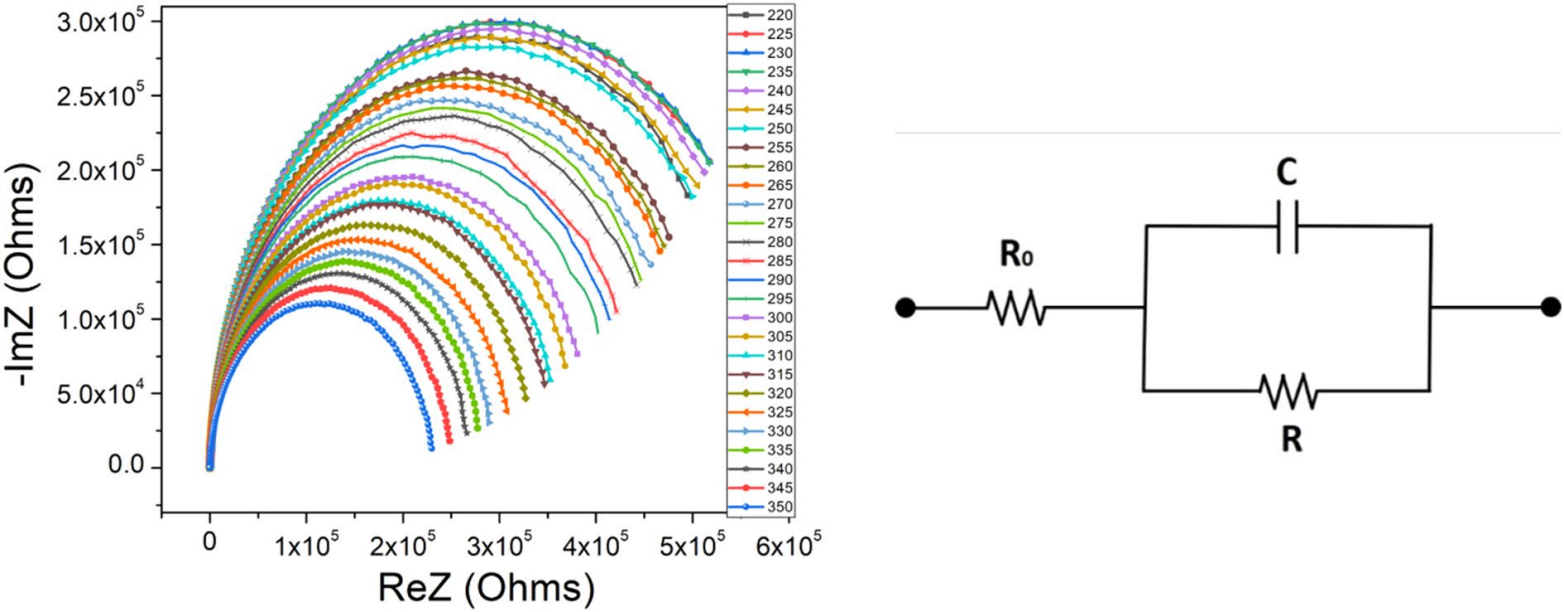
The impedance spectra (left) and the equivalent circuit diagram of *n*CdS/*p*ZnTe HJ (right).

The method of impedance spectroscopy (IS) allows determining the behavior of the polarization conductivity. In cartesian coordinates, the impedance can be expressed as Z(ν) = ReZ(ν)–ImZ(ν) or Z = Z′–*j*Z″, where ReZ(ν) (or Z′) represents the real part and ImZ(ν) (or Z″) represents the imaginary part of the impedance. Typically, in this type of research, a frequency region between 10 Hz and 10 MHz is used. The Cole–Cole (or Nyquist) diagram, which shows the dependence of the imaginary (Z″) versus the real (Z′) component of impedance Z, is plotted for a circuit containing a capacitor and a resistor in parallel. This dependence for a capacitor-resistor parallel circuit represents a semicircle in the fourth quadrant, with the origin on the real axis and a radius of R/2. If the semicircle does not start from the origin, this indicates the presence of series resistance in the studied structure. The Bode plot shows the dependence of ln|*Z*| of ln*ω* for a circuit containing a capacitor and a resistor in parallel, illustrating the influence of frequency on impedance. Figure 13 (left) presents the real Z′ and imaginary Z″ components of the complex impedance Z = Z′–*i*Z″ (where *i* = − 1) dependencies for the *n*CdS/*p*ZnTe HJ, in the frequency region 100 Hz–3 MHz, at various temperatures.

Complex impedance measurements of the sample provide useful information about the real component Z′ (the resistive part) and the imaginary component Z″ (the reactive part) of the complex impedance. It’s known that in the case of a capacitance sample inserted into a variable electromagnetic field of frequency ω, the equivalent circuit diagram^30^ is represented as in Fig. 13 (right). According to the circuit diagram, the real component (Z′) is:

22 $$Z^{\prime} = R_{1} + \frac{R}{{1 + \omega^{2} C^{2} R^{2} }}.$$

And the imaginary component (Z″)

23 $$Z^{\prime\prime} = {\text{R}}_{1} + \frac{{\omega {\text{CR}}^{2} }}{{1 + \omega^{2} {\text{C}}^{2} {\text{R}}^{2} }}.$$

Eliminating the frequency from Eqs. (22) and (23), we obtain:

24 $$\left( {Z^{\prime} - {\text{R}}_{1} - \frac{{\text{R}}}{2}} \right)^{2} + Z^{\prime 2} = \left( {\frac{{\text{R}}}{2}} \right)^{2} .$$

**Figure 14:**
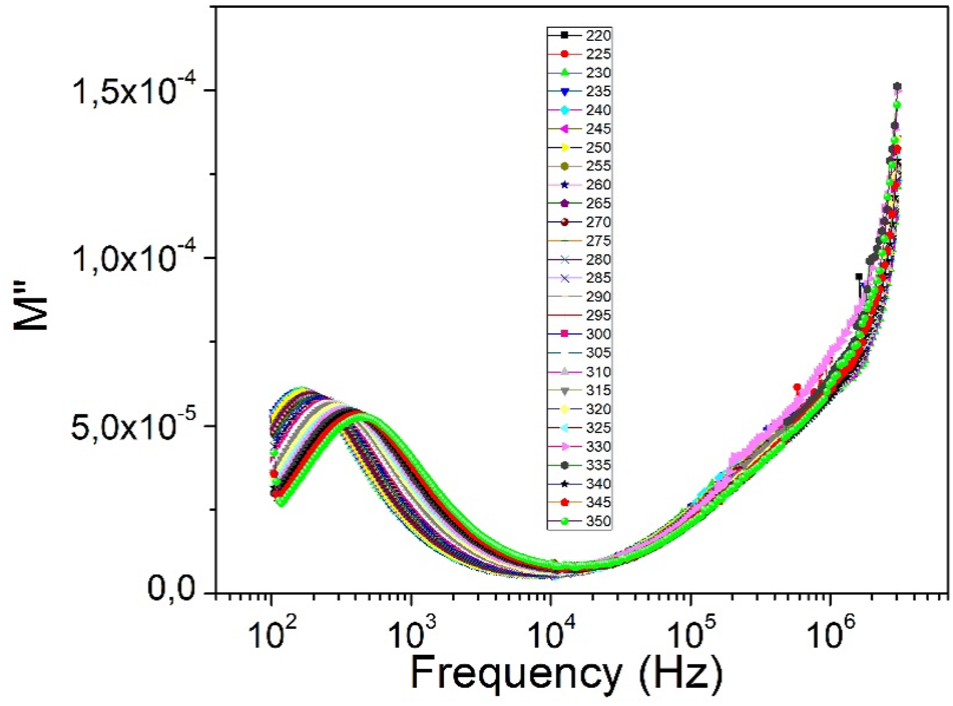
The complex impedance modulus-frequency dependencies of *n*CdS/*p*ZnTe HJ.

The equation of the circle with center at *C*(*R*_1_ + *R*/2; 0). The radius *r* = *R*/2 was used to estimate the parameters of the equivalent circuit. The resistance *R* can be determined from the diameter of the semicircle, and the capacitance *C* can be calculated from the frequency corresponding to the maximum value of the semicircle. The complex electrical conductivity of the sample contains the components *σ'* and *σ''*, which represent the real and imaginary part of the complex electrical conductivity. They can be expressed by the relations:

25 $$\sigma^{\prime} = \frac{\rho ^{\prime}}{M},$$

and

26 $$\sigma^{\prime\prime} = \frac{{\rho^{\prime\prime}}}{M},$$

where M represents the modulus of the impedance, defined by the relation:

27 $${\text{M}} = \left| {{\text{Z}}^{*} } \right|^{2} \cdot \left( {\frac{{\text{A}}}{{\text{d}}}} \right)^{2} ,$$

where ${Z}^{*}=\sqrt{{Z{\prime}}^{2}+{Z}^{*2}}$ represents the impedance modulus of the *n*CdS/*p*ZnTe HJ. The impedance modulus exhibits peaks at low frequencies. The low frequency peak suggests that ions can move over long distances whereas high frequency peak supports the confinement of ions in their potential well^31^. The impedance modulus of the *n*CdS/*p*ZnTe HJ shown in Fig. 14 indicates the low frequency peak. The observed peak broadening indicates the spread of relaxation time with different time constants which supports the non-Debye type of relaxation in the materials. We found that M″ exhibits a maximum that shifts to a higher frequency with increasing temperature, indicating a correlation between the motions of mobile ionic charges. The frequency region where the peaks occur is indicative of transition from long range to short range mobility of the charge carriers^32,33^.

Such results may possibly be related to a lack of restoring force governing the mobility of the charge carriers under the action of an induced electric field. This behavior supports that as the frequency increases, each ion moves a shorter and shorter path of electric field, until the electric field changes so rapidly that the ions only rattle within the confinement of their potential energy wells and the ion can make only the localized motion within the wells. Since the most interesting parameter is the relaxation time, we plotted the temperature dependence τ(T) in the whole analyzed temperature range. The relationship between the maximum frequency *ω*_*max*_ at which the imaginary component Z″ has a maximum is described by the equation^34^:

28 $$2\pi \omega_{\max } \tau = 1.$$

Based on the above considerations, it can be concluded that the polarization conductivity at higher frequencies behavior matches the Vogel–Fulcher law interfacial polarization. Using the experimental values of the frequency *ω*_*max*_, the relaxation time *τ* was estimated using Vogel–Fulcher equation:

29 $$\tau = \tau_{0} {\text{exp}}\left( {\frac{{E_{A} }}{{{\text{k}}_{{\text{B}}} \left( {{\text{T}} - {\text{T}}_{{{\text{VF}}}} } \right)}}} \right),$$

that also implemented to calculate the activation energy due to the variation in the relaxation time *τ, τ*_*0*_ is the pre-exponential factor, *E*_*A*_ is the activation energy, *T*_*VF*_ is the Vogel–Fulcher (VF) temperature and *k*_*B*_ is the Boltzmann constant. *T*_*VF*_ being a singular divergence temperature is usually regarded as the freezing temperature of a freezing phenomenon. The *E*_*A*_ denotes the non-Arrhenius activation energy and *τ*_0_ defines the relaxation time at high temperatures.

**Figure 15:**
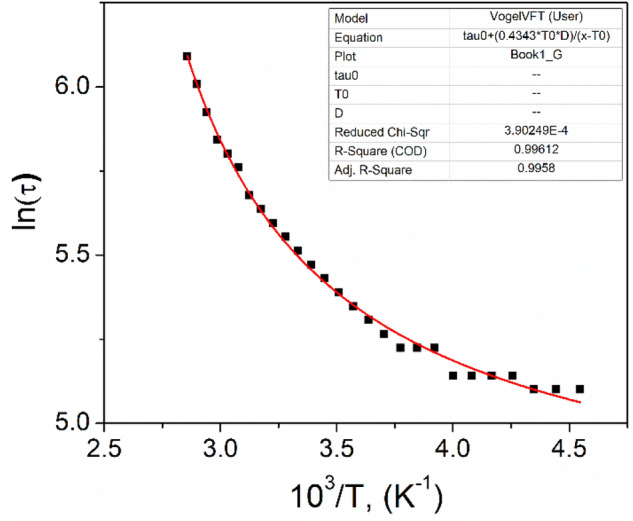
The relaxation time–temperature dependence of *n*CdS/*p*ZnTe HJ.

A non-linear relationship between the relaxation time–temperature dependence can be observed in Fig. 15. According to Josher’s power law for AC conductivity, formula (20), for the polarization conductivity, at temperature higher than zero value of *T*_*VF*_ parameter from (29), the exponent *s* has a value more than one, that means faster rise of polarization conductivity with temperature compared with the Arrhenius relation. Higher than zero value of *T*_*VF*_ parameter shows the faster rise of *τ*(*T*) on temperature compared to the Arrhenius relation. If the material exhibits behavior that fits the Vogel–Fulcher law, this indicates that the material has a disordered nature and is likely an ionic conductor. Conductivity is influenced by the movement of ions in the material. The conductivity described by the VF law can be influenced by the presence of trapping states in the material. These states can serve as centers for recombination or generation of charge carriers. There may also be energy levels that serve as barriers to ion movement, contributing to the observed relaxation time–temperature dependence. It is possible that interface or surface states contribute to the same behavior observed in both capacitance and conductivity measurements.

So, we can conclude that different transport mechanisms become dominant in *n*CdS/*p*ZnTe HJ at different temperatures regions, each with a different activation energy, respectively. This can be attributed to the fact that the bulk conductivity estimates are derived from low frequencies than those used in the calculation of relaxation time, which is determined by the frequency at which tanδ reaches its maximum. Despite this difference, the estimated values are comparable, indicating a similar origin for both processes.

Generally, in nCdS/pZnTe HJ, where both processes are present, the electron distribution is described by the density matrix, which includes both the quantum uncertainty of the position due to tunneling and the classical uncertainty of the position due to stochastic hops, even if this process is not "phonon-assisted" the interpretation of hops still remains classic/incoherent, since the theory assume that the total probability of the sequence of hops is the product of probabilities, not the amplitudes.

## Conclusions

The development of the *n*CdS/*p*ZnTe HJ using the CSS method has been successfully achieved. The characterization of these cells revealed several important features:

- The J–V–T and C–V–T characteristics display a complex behavior. The forward bias current is dominated by thermionic emission and recombination at all temperatures with domination of the recombination component via interface states and traps intersecting the space-charge region at high temperatures.
- The experimental values of the dielectric constant, dielectric losses, and the imaginary part of the electric modulus were found to be strong dependent on frequency and temperature.
- The C–F measurements indicate the presence of multiple dielectric relaxation processes within the heterostructure, contributing to the observed complex behavior of dielectric losses. As frequency increases, dielectric losses generally decrease at all measurement temperatures. Beyond approximately 100 kHz, there is a noticeable reversal in the trend, the dielectric losses starting to increase again.
- The total conductivity is the sum of the frequency independent σ_dc_ and frequency dependent σ_ac_(ω) conductivities. The frequency-dependent conductivity values follow Jonsher's power law, which means that the dielectric relaxation is due to the mobile electric charge carriers. When a mobile charge carrier jump from their original position to a new site, they will be in a state between two potential energy minima. If *s* < 1, the movement of charge carrier is translational and if* s* > 1, the movement of charge carrier is localized.

So, the interplay between the ionic conductivity and the interfacial polarization is a key point for understanding current mechanism in the *n*CdS/*p*ZnTe HJ.

### Supplementary Information

Supplementary Information.

## Data Availability

All data generated or analyzed during this study are included in this published article [and its supplementary information files].
