# Author Correction: DC current–voltage and impedance spectroscopy characterization of *n*CdS/*p*ZnTe HJ

**DOI:** 10.1038/s41598-024-66982-2

**Published:** 2024-07-12

**Authors:** I. Lungu, R. E. Patru, A. C. Galca, L. Pintilie, T. Potlog

**Affiliations:** 1 Laboratory of Organic/Inorganic Materials for Optoelectronics, Moldova State University, 2009 Chisinau, Republic of Moldova; 2 Complex Heterostructures and Multifunctional Materials Laboratory, National Institute of Materials Physics, 077125 Magurele, Ilfov Romania

Correction to: *Scientific Reports* 10.1038/s41598-024-63615-6, published online 5 June 2024

The Acknowledgements section in the original version of this Article was incorrect.

“This work was supported by the Ministry of Education and Research of Republic of Moldova, research grant #011209 and OPERA • COST ACTION CA20116 European Network for Innovative and Advanced Epitaxy. NIMP authors kindly acknowledge the financial support of the Romanian Ministry of Research, Innovation and Digitalization in the framework of the UEFISCDI PN-III-P4-ID-PCE-2020-0827 (contract no. PCE74/09.02.2021) and Core Program Project PC3-PN23080303.”

now reads:

“This article/publication is based upon work from COST Action OPERA - European Network for Innovative and Advanced Epitaxy – CA20116, supported by COST (European Cooperation in Science and Technology, www.cost.eu). Also, the authors thanked the Ministry of Education and Research of Republic of Moldova for financial support for grant #011209 NIMP and kindly acknowledge the financial support of the Romanian Ministry of Research, Innovation and Digitalization in the framework of the UEFISCDI PN-III-P4-ID-PCE-2020-0827 (contract no. PCE74/09.02.2021) and Core Program Project PC3-PN23080303."

The original Article has been corrected.

