# Supplementary material for: DC current–voltage and impedance spectroscopy characterization of nCdS/pZnTe HJ
Source: Sci Rep. 2024 Jun 5;14:12955. doi: 10.1038/s41598-024-63615-6 (PMC11153606; doi:10.1038/s41598-024-63615-6)
Supplement: Supplementary file 1 — Supplementary Information. [file 41598_2024_63615_MOESM1_ESM.docx]

**SUPPLEMENTARY INFORMATION**

**DC Current-Voltage and Impedance Spectroscopy Characterization of *n*CdS/*p*ZnTe HJ**

*I. Lungu^1^*, R. E. Patru^2^, A. C. Galca^2^, L. Pintilie^2^ and T. Potlog^1^*

*^1^ Organic/Inorganic Materials in Optoelectronics, Moldova State University, MD-2009, Chisinau, Republic of Moldova*

*^2^ Complex Heterostructures and Multifunctional Materials Laboratory, National Institute of Materials Physics, RO-077125, Magurele, Ilfov, Romania*





**Figure S1.** The conductivity-temperature dependence of the *n*CdS/*p*ZnTe HJ

Electrical conductivity values were determined from the data analysis and were plotted as a function of temperature. The ln σ_dc_ versus 1000 (T −1) plot shows that the Arrhenius model is not fully applicable for explaining the conductivity mechanism of CdS/ZnTe HJ. For the low temperature interval, the conductivity is best studied by VRH (Variable range hopping) model which follows Mott equation:


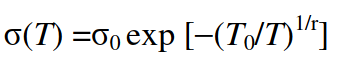


where T_0_ and σ_0_ are Mott characteristic temperature and conductivity (at T=∞) and exponent *r* is the dimensionality factor having values 2, 3, 4 for 1-dimension, 2-dimension and 3-dimension conduction mechanism, respectively.

**Figure S2.** Dependence ln σ versus T^−1/4^ for *n*CdS/*p*ZnTe HJ

The plot logσ_dc_ as versus of T^−1/4^ gives the values of Mott characteristic temperatureT_0_ and σ_0_ (conductivity at T=∞) as:


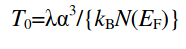


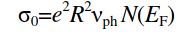


where R=[9/{8παk_B_TN(E_F_)}]^1/4^ is the average hopping distance , α^−1^ is the localization length, λ (-18.1) is the dimensionless constant, N(E_F_) is the density state at the Fermi level and ν_ph_ is the phonon frequency (10^13^ Hz). It is observed that the conductivity data fits for the VRH model with r=4 having the linearity factor of 0.903 is suitable for explaining the conduction mechanism wherein the charge transport occurs by phonon assisted hopping or by thermally stimulated jumps between the localized sites.

Below 260 K, the conductivity data deviate from the linear behavior because in low temperature region charge conduction is mainly dominated by the thermally stimulated tunneling through the localized sites. Therefore, the observed dc conductivity is contributed as the sum of hopping conduction and tunneling conduction.

**
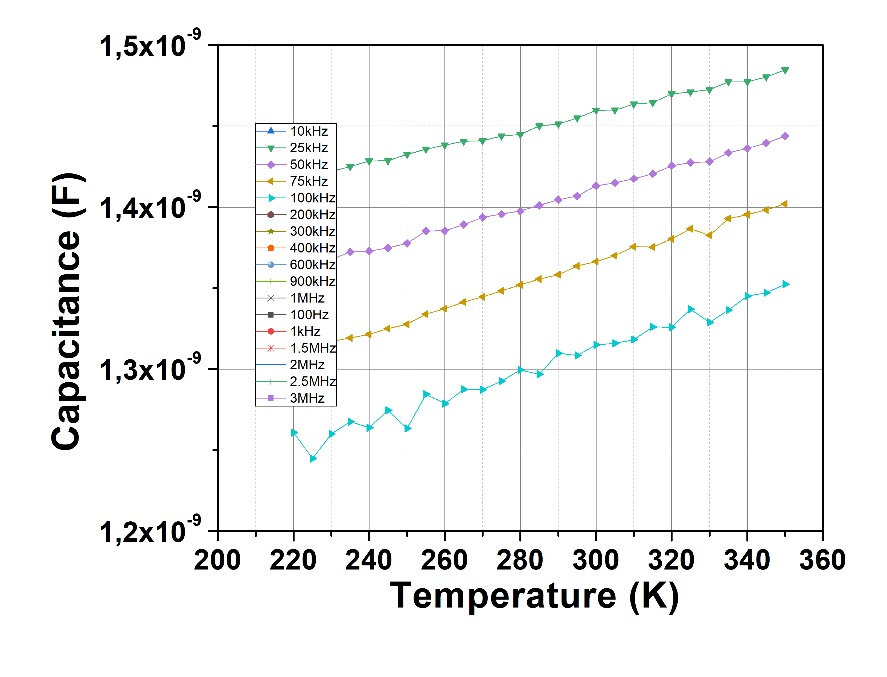
**

**Figure S3.** The capacity-temperature dependencies of the *n^+^*CdS/*p*ZnTe heterojunction at different measurement frequencies.

This change is due to the fact that the CdS and ZnTe used in the capacitance are sensitive to temperature variations, causing its properties to change. The accumulation of additional charge carriers at the interface states led to excess capacitance at higher temperature. The reason for the dominant increase in capacitance is explained by model of charge carriers blocked at the

electrodes. Additionally, the formed space charge layer also leads to a large increase in capacitance value.

**Figure S4.** The 1/C ^2^ =f(U) curves of *n*CdS/*p*ZnTe HJ for different temperatures

**Table S1.** Electrical parameters estimated from C-V characteristics at different temperatures

| **T, K** | **N_ef_ * 10^14^** | **V_bi_, V** |
| --- | --- | --- |
| 220 | 2.8 | 0.96 |
| 300 | 0.6 | 1.02 |
| 350 | 0.5 | 1.0 |

**Figure S5.** The temperatures dielectric loss dependencies of the nCdS/pZnTe heterojunction for different measurement frequencies

The dielectric losses of CdS/ZnTe increased at low temperatures and decreased at high temperatures as the frequency increased. This may be attributed to the transition of the dominant component of dielectric loss from relaxation polarization loss to conductive loss at higher temperatures.

**Figure S6.** The relaxation time-temperature dependence of n^+^CdS/pZnTe heterojunction.

From above it is clear that resistance of the equivalent circuit of CdS/ZnTe HJ is inversely proportional to relaxation time and relaxation time is inversely proportional to temperature. Therefore, if the temperature increases, then relaxation time decreases. The temperature dependence of τ is obviously subject to the Arrhenius thermal activation.

**The XRD analysis** **of AZO, AZO/CdS, ZnTe and for AZO/CdS/ZnTe/Ag**

**Figure S7.** X-ray diffractogram of of AZO, AZO/CdS, ZnTe thin films

**Figure S8.** X-ray diffractogram of of AZO/CdS/ZnTe HJ

**Table S2.** The structural parameters of AZO, AZO/CdS, ZnTe and AZO/CdS/ZnTe/Ag.

| **Samples** | **faza** | **Exp** | | **Ref** | | **Error**  **%** | **D, nm** | **𝜀*10^-3^** |
| --- | --- | --- | --- | --- | --- | --- | --- | --- |
|  |  | **a, A** | **c, A** | **a, A** | **c, A** |  |  |  |
| **AZO** | ZnO – h | - | 5.2242 | 3.249 | 5.205 | 0.37 | 10.08 | 4.29 |
| **AZO/CdS** | ZnO – h | - | 5.2182 | 3.249 | 5.205 | 0.25 | 8.41 | 21.10 |
|  | CdS – h | 4.3168 | 6.7268 | 4.141 | 6.720 | (a) 4.25  (c) 0.10 | 10.91 | 0.64 |
| **ZnTe** | ZnTe – c | 6.1013 | 6.1013 | 6.103 | 6.103 | 0.03 | 27.43 | 1.10 |
| **AZO/CdS/**  **ZnTe/Ag** | ZnTe – c | 6.1027 | 6.1027 | 6.103 | 6.103 | 0.005 | 68.96 | 4.17 |
|  | ZnO - h | - | 5.2126 | 3.249 | 5.205 | 0.15 | n/p | n/p |
|  | CdS – h | - | 6.6892 | 4.141 | 6.720 | 0.46 | 59.11 | 5.00 |
|  | CdS -h |  |  | 4.137 | 6.716 | 0.40 |  |  |
|  | Ag – c | 4.0809 | 4.0809 | 4.086 | 4.086 | 0.12 | 290.80 | 3.05 |

# **Table S3.** Microstructural parameters obtained from Scherrer's method

| Samples | 2𝜃 | d. A | FWHM.  rad | D. A | 𝜀 |  |  | h | k | l |
| --- | --- | --- | --- | --- | --- | --- | --- | --- | --- | --- |
| AZO | 34.32 | 2.6121 | 0.0066 | 244.1 | 0.0054 | AZO | h | 0 | 0 | 2 |
|  | 47.46 | 1.9150 | 0.0506 | 33.26 | 0.0288 | AZO | h | 1 | 0 | 2 |
|  | 62.73 | 1.4807 | 0.0133 | 135.3 | 0.0055 | AZO | h | 1 | 0 | 3 |
| -- | -- | -- | -- | -- | -- |  |  |  |  |  |
| AZO/CdS |  |  |  |  |  |  |  |  |  |  |
|  | 26.49 | 3.3634 | 0.0040 | 399.3 | 0.0042 | CdS | h | 0 | 0 | 2 |
|  | 34.36 | 2.6091 | 0.0050 | 322.4 | 0.0041 | AZO | h | 0 | 0 | 2 |
|  | 43.54 | 2.0777 | 0.0326 | 50.8 | 0.0204 | CdS | h | 1 | 1 | 0 |
|  | 47.48 | 1.9143 | 0.0246 | 68.4 | 0.0140 | AZO | h | 1 | 0 | 2 |
|  | 54.55 | 1.6816 | 0.0071 | 245.4 | 0.0034 | CdS | h | 0 | 0 | 4 |
|  | 62.72 | 1.4807 | 0.0282 | 63.9 | 0.0116 | AZO | h | 1 | 0 | 3 |
|  | 72.43 | 1.3044 | 0.0104 | 183.7 | 0.0036 | CdS | h | 1 | 1 | 4 |
| -- | -- | -- | -- | -- | -- |  |  |  |  |  |
| ZnTe | 25.26 | 3.5248 | 0.0023 | 673.8 | 0.0026 | ZnTe | c | 1 | 1 | 1 |
|  | 29.23 | 3.0542 | 0.0029 | 543.5 | 0.0028 | ZnTe | c | 2 | 0 | 0 |
|  | 41.81 | 2.1600 | 0.0024 | 694.2 | 0.0016 | ZnTe | c | 2 | 2 | 0 |
|  | 49.47 | 1.8419 | 0.0023 | 737.1 | 0.0013 | ZnTe | c | 3 | 1 | 1 |
|  | 51.83 | 1.7632 | 0.0018 | 958.8 | 0.0009 | ZnTe | c | 2 | 2 | 2 |
|  | 60.61 | 1.5272 | 0.0028 | 647.7 | 0.0012 | ZnTe | c | 4 | 0 | 0 |
|  | 66.73 | 1.4012 | 0.0025 | 740.0 | 0.0009 | ZnTe | c | 3 | 3 | 1 |
|  | 68.70 | 1.3657 | 0.0042 | 449.0 | 0.0015 | ZnTe | c | 4 | 2 | 0 |
| -- | 76.36 | 1.2468 | 0.0027 | 734.1 | 0.0008 | ZnTe | c | 4 | 2 | 2 |
| AZO/CdS/  ZnTe/Ag | 25.29 | 3.5210 | 0.0022 | 709.0 | 0.0025 | ZnTe | c | 1 | 1 | 1 |
|  | 26.64 | 3.3455 | 0.0044 | 363.0 | 0.0046 | CdS | h | 0 | 0 | 2 |
|  | 29.27 | 3.0503 | 0.0019 | 861.3 | 0.0018 | ZnTe | c | 2 | 0 | 0 |
|  | 34.40 | 2.6063 | 0.0046 | 351.4 | 0.0037 | AZO | h | 0 | 0 | 2 |
|  | 38.20 | 2.3550 | 0.0043 | 381.1 | 0.0031 | Ag | c | 1 | 1 | 1 |
|  | 41.86 | 2.1574 | 0.0016 | 1045.2 | 0.0010 | ZnTe | c | 2 | 2 | 0 |
|  | 44.38 | 2.0405 | 0.0065 | 257.6 | 0.0040 | Ag | c | 2 | 0 | 0 |
|  | 49.51 | 1.8404 | 0.0016 | 1087.7 | 0.0008 | ZnTe | c | 3 | 1 | 1 |
|  | 51.90 | 1.7613 | 0.0182 | 94.1 | 0.0094 | ZnTe | c | 2 | 2 | 2 |
|  | 54.88 | 1.6723 | 0.0218 | 79.7 | 0.0105 | CdS | h | 0 | 0 | 4 |
|  | 60.65 | 1.5264 | 0.0021 | 843.3 | 0.0009 | ZnTe | c | 4 | 0 | 0 |
|  | 64.56 | 1.4431 | 0.0070 | 258.8 | 0.0028 | Ag | c | 2 | 2 | 0 |
|  | 66.77 | 1.4004 | 0.0022 | 845.9 | 0.0008 | ZnTe | c | 3 | 3 | 1 |
|  | 72.46 | 1.3039 | 0.0116 | 165.4 | 0.0039 | CdS | h | 1 | 1 | 4 |
|  | 76.39 | 1.2463 | 0.0155 | 126.6 | 0.0049 | ZnTe | c | 4 | 2 | 2 |
|  | 77.53 | 1.2308 | 0.0112 | 175.8 | 0.0035 | Ag | c | 3 | 1 | 1 |
